# Mitigating the Negative Mental Health Impact of Racism on Black Adolescents—A Preventive Perspective

**DOI:** 10.1001/jamanetworkopen.2023.40577

**Published:** 2023-11-01

**Authors:** Kevin M. Simon

**Affiliations:** Department of Psychiatry and Behavioral Sciences, Boston Children’s Hospital, Boston, Massachusetts; Division of Addiction Medicine, Boston Children’s Hospital, Boston, Massachusetts; Department of Psychiatry, Harvard Medical School, Boston, Massachusetts; Center for Behavioral Health and Wellness, Boston Public Health Commission, Boston, Massachusetts.

Racism is a public health crisis with adverse mental health impacts that affect youths and endure from adolescence through adulthood.^1^ The intricate fabric of racism in the United States represents a multilevel, multidimensional, and pervasive context that systematically engenders racial disparities across generations and throughout individuals’ lives.^2^ As a firmly entrenched element of our society, racism goes beyond isolated incidents of bias to infuse the systems and structures that define everyday life. The adverse impacts of racism, particularly on mental health among Black adolescents, are devastating and widespread.^3^ The link between racial discrimination and negative mental health outcomes, including depression, has been well established by a vast body of research.^4^

In this growing awareness era, deepening our understanding of these implications and developing effective preventive interventions is critical. Herein lies the importance of the study by Kogan et al.^5^ The study by Kogan et al^5^ rigorously demonstrates the positive mental health impact that a culturally tailored prevention program, Strong African American Families (SAAF), can have on Black adolescents through a secondary analysis focused on moderation effects. SAAF is a 7-session program targeting youth aged 10 to 14 years and their caregivers.^5^ SAAF aims to capitalize on the strength of Black families, support parents and children during the pivotal transition to teen years, and ultimately prevent substance use.^5^

The data in the study by Kogan et al^5^ came from a randomized prevention trial, conducted between June 2013 and June 2019, that included 472 Black adolescents and their primary caregivers from 7 rural counties in Georgia. Participants were drawn from lists of Black fifth-grade students in regional public schools. The post hoc analysis by Kogan et al^5^ found that exposure to racial discrimination at age 13 years was associated with increased depressive symptoms at age 14 years. The researchers’ findings contribute to the evolving body of evidence emphasizing the significant mental health burden that racial discrimination places on Black youth.^6,7^ The multigroup analyses by Kogan et al^5^ indicated that experimental conditions significantly moderated the association of racial discrimination with depressive symptoms.

While the original SAAF study was meticulously conducted with a broad demographic reach, its primary focus was on a specific subset: rural US Black adolescents. As a result, the post-hoc analysis by Kogan et al^5^ carries this inherent limitation. This means that their findings might not fully apply to urban Black individuals or other racial and ethnic groups. Nonetheless, the research offers significant insight into the effects of racial discrimination on Black adolescents’ mental health.^5^

Placing this study by Kogan et al^5^ in the context of earlier research and the current state of science on racial discrimination and mental health, it becomes evident how it complements and expands on prior work. For example, a study by Seaton and Linda^4^ explored the association of daily exposure to racial discrimination with racial identity among Black youth and found a strong association, but their study did not delve into potential mitigation strategies. In contrast, Kogan et al^5^ not only substantiate this association but also venture further by evaluating the effect of a preventative intervention program, providing a potential solution for the mental health challenges Black adolescents face due to racial discrimination. The study by Kogan et al^5^ builds on the understanding that racial discrimination negatively affects Black adolescents’ mental health, a notion previously established, and illuminates a pathway for mitigating this impact.

The unique focus of the study by Kogan et al^5^ on rural Black adolescents and its demonstration of a positive preventive intervention makes it a standout contribution to the existing literature. It aligns with prior research and builds on it by introducing a novel aspect of inquiry. In this way, the study by Kogan et al^5^ lays a foundation for future research to generalize these findings for a broader, historically marginalized, and undersupported demographic.

The study by Kogan et al^5^ further complements the American Academy of Pediatrics’ landmark policy statement, “The Impact of Racism on Child and Adolescent Health,” which describes the profound effects of racism on general health by providing empirical evidence of the specific mental health impact on Black adolescents.^1^ By providing empirical evidence of specific mental health impacts on Black adolescents, this study by Kogan et al adds value.^5^ It highlights the effectiveness of a culturally sensitive prevention program—SAAF—implemented by trained Black facilitators. In doing so, Kogan et al^5^ advance the conversation from problem identification to solution exploration.

The implications of this study for clinical practice and policy are substantial. The evidence that racial discrimination experienced at age 13 years was associated with increased depressive symptoms by age 14 years underscores the importance of proactive mental health care strategies for Black adolescents. These strategies include early mental health screenings and effective interventions to address known challenges.

When considering the broader community from which the participants in the study by Kogan et al^5^ originate, the impacts of racial discrimination extend beyond individual mental health, seeping into entire communities and significantly influencing public health. By focusing on the mental health toll of racial discrimination on Black adolescents, this study brings a pressing public health issue into sharp focus.

Moreover, the findings in the study by Kogan et al^5^ underscore the pivotal role of prevention programs focusing on racial identity, racial socialization processes, and parenting behavior. Such initiatives could alleviate the mental health impacts of racial discrimination, foster positive coping mechanisms, and prevent the internalization of harmful narratives related to racial inferiority.

In conclusion, this study by Kogan et al^5^ provides a comprehensive look into the mental health consequences of racial discrimination among Black adolescents in the US. By situating the study within broader clinical and community contexts, it becomes clear that addressing the mental health effects of racial discrimination demands urgent systemic changes and preventative measures. This study by Kogan et al^5^ is a poignant reminder of the critical role that academic communities play in opposing the mental health effects of racial discrimination.
